# Molecular Docking Study for Binding Affinity of 2*H*-thiopyrano[2,3-*b*]quinoline Derivatives against CB1a

**DOI:** 10.1155/2023/1618082

**Published:** 2023-01-09

**Authors:** Shivangi Sharma, Shivendra Singh

**Affiliations:** Department of Applied Chemistry, Amity School of Applied Sciences, Amity University Madhya Pradesh, Gwalior, Madhya Pradesh 474005, India

## Abstract

Quinoline-based molecules are major constituents in natural products, active pharmacophores, and have excellent biological activities. Using 2*H*-thiopyrano[2,3-*b*]quinoline derivatives and CB1a protein (PDB ID: 2IGR), the molecular docking study has been revealed in this article. The study of *in silico* molecular docking analysis of such derivatives to determine the binding affinity, residual interaction, and hydrogen bonding of several 2*H*-thiopyrano[2,3-*b*]quinolines against CB1a is reported here. The current work demonstrated that 2*H*-thiopyrano[2,3-*b*]quinoline derivatives could be effective antitumor agents to produce potent anticancer medicines in the near future.

## 1. Introduction

Cancer is marked by the uncontrolled, rapid, and pathological growth of improperly altered cells and used in a broader sense. Despite considerable advancements in cancer therapy in recent times, cancer remains the world's second biggest cause of death, trailing only cardiovascular (CVS) diseases [1]. Chemotherapeutic drug resistance is still a major issue in the fight against cancer; in addition, chemotherapy is further hampered by a lack of selectivity. Anticancer medications, in general, kill both healthy and cancerous cells, and they frequently have major side effects. Many efforts have been undertaken to find new chemotherapeutic medicines with low side effects and to develop safe and effective strategies to treat this disease [2]. Among all cancer types, cervical cancer has the world's fourth most frequent malignancy among women, with the third highest fatality rate [3]. Cervical cancer alone accounts for about 12% of all cancers in women worldwide, according to a WHO (World Health Organization) report [4], and it is more common in developing nations. Until now, chemotherapeutic medication treatment for cervical cancer has been associated with a poor prognosis and a slew of negative side effects [5]. Therefore, scientists are working for the development of new safer chemicals having potent anticancer activity. For the identification of efficient cellular targets [6], early detection is required which improves the efficacy of the available chemotherapeutic approaches in the field of cancer research [7, 8].

**Figure 1:**
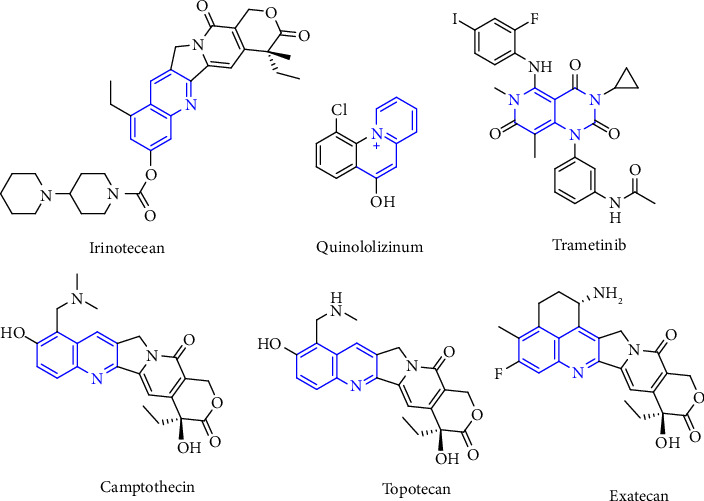
Some quinoline based anticancer drug molecules.

A variety of substituted quinoline derivatives are widely known for their crucial role in the design of innovative pharmacological moieties for therapeutic use, as evidenced by the large number of commercially available medications that include this heterocycle [9]. Quinoline compounds are pharmacologically important and have piqued the interest of chemists and biologists alike due to their wide range of biological actions [10]. Quinoline entities are fundamental building blocks for many naturally occurring chemicals; particularly, bioactive quinoline heterocycles are found in alkaloids of quinoline derived from diverse plant families such as Fumariaceae, Papavaraceae, Rutaceae, and Berberidaceae [10, 11]. Many writers have reviewed the wide range of biological actions of quinoline compounds [12]. Insecticidal [13], antimicrobial [14], antimalarial [15], antiamoebic [16], analgesic [17], vasorelaxing [18], antidiabetic [19], antimycobacterial [20], anticancer [21], anti-inflammatory [22], antihypertensive [23], antiulcer [24], and anti-HIV (human immunodeficiency virus) [25].  Figure 1 shows some clinically used pharmacological based compounds having quinoline heterocyclic core.

The quinoline core is a significant structural unit in many naturally occurring compounds [26–29], with interesting biological activity, and many conventional medications. Quinoline core having substitutions at different positions has shown substantial anticancer efficacy against a variety of targets, including topoisomerase I [30], tubulin [31], protein kinase [32], and so on. Some well-known quinolone-based anticancer medicines have been approved by the FDA (Food and Drug Administration) e.g., camptothecin, irinotecan, topotecan, and others. Furthermore, few such quinoline-based anticancer drugs are still in clinical testing phase such as bosutinib, lenvatinib, cabozantinib, and farnesyltransferase inhibitors (tipifarnib) [33].

Several well-known compounds having 2*H*-thiopyrano[2,3-*b*]quinolines can be synthesized in a highly efficient manner and create one C-S and one C-C bond with a high extraction economy, likely via a Michael addition, intramolecular aldol-dehydration domino process. It is common knowledge that combining pharmacophore units with different biological activities results in a new hybrid entity with higher biological processes and efficacy than the parent medications. Based on the previous discoveries, unique bioactive hybrid compounds based on quinoline having better anticancer efficacy have been developed nowadays.

**Figure 2:**
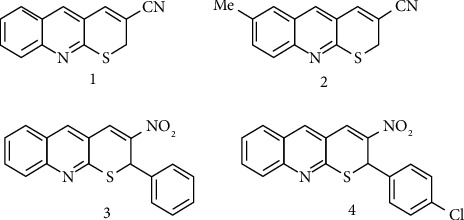
Structure of some compounds having 2*H*-thiopyrano[2,3-*b*]quinoline cores.

In this article, we have reported the binding affinity and several interactions of compounds **1**–**4** (as shown in Figure 2) with the anticancer-based protein CB1a (CB1a, a novel anticancer peptide derived from natural antimicrobial peptide cecropin B: **PDB ID: 2IGR**). The values of binding affinity for **1–4** lies between −**5.3 and** −**6.1 Kcal/mol**. The interaction also shows that compound **4** shows highest binding affinity (−**6.1 Kcal/mol**) as compared to other derivatives of thiopyrano[2,3-*b*]quinolines. For the docking study, we used several software packages such as AutoDock vina 4, discovery studio, and protein-ligand interaction profiler. These software packages help in the study and analysis of docking position, docking size, binding affinity, energy range as well as exhaustiveness. Several amino acids are attached with ligands (thiopyrano[2,3-*b*]quinoline), and its analogues show several interactions; some are as follows: ILE A-8, ILE A-18, LYS A-7, LYS A-8, LYS A-10, LYS A-11, LYS A-13, LYS A-16, LYS A-25, VAL A-14, LYS A-16, LYS A-26, PHE A-15, TRP A-12, TRP A-25, LYS A-10, and GLU A-9.

## 2. Materials and Methods

Synthesis of Thiopyrano[2,3-b]quinolines.

Meth Cohn et al. synthesized 2-chloro-3-carbaldehyde quinoline derivatives in high yield by using acetanilide using phosphoryl chloride solution under the presence of dimethyl formamide (DMF) at 70–80°C [34].

**Scheme 1:**
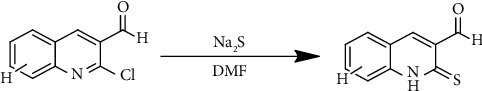
Substitution reaction on 2-chloro-3-formyl quinoline.

Srivastava and Singh et al. synthesized 3-formyl-quinoline-2-thione derivative by using 2-chloro-3-carbaldehydes quinoline derivative using sodium sulphide (Na_2_S) in the presence of dimethyl formamide (DMF) solvent at room temperature (Scheme 1) [35].

**Scheme 2:**
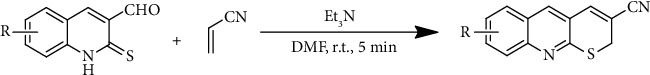
Synthesis of 3-cyanothiopyrano[2,3-*b*]quinoline derivatives.

Singh et al. revealed the synthesis of thiopyrano[2,3-(b)]quinoline derivatives by using 3-formyl-quinoline-2-thione derivative and acrylonitrile in the presence of cheap base triethylamine (TEA) and dimethyl formamide (DMF) at room temperature [36]. It is a rapid and efficient one-pot reaction for the synthesis of 3-cyanothiopyrano[2,3-(b)]quinoline derivatives (Scheme 2). The whole reaction was synthesized very easy by the Domino Michael addition followed by cyclization.

**Scheme 3:**
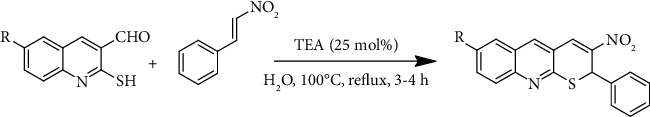
Synthesis of 3-nitrothiopyrano[2,3-*b*]quinoline derivatives.

Kumar et al. demonstrated the synthesis of 3-nitro-2-phenyl-2*H*-thiopyrano[2,3-*b*] quinoline derivatives using 2-mercaptoquinoline-3-carbaldehyde and substituted trans-*β*-nitrostyrenes in presence of triethylamine (TEA) at 100°C (Scheme 3) [37].

## 3. Molecular Docking Studies

Cecropins are a category of antimicrobial peptides abundantly available in Hyalophora cecropia's immunological hemolymph. The *N*-terminal regions of native cecropins include basic residues, while the C-terminal parts have hydrophobic residues. Cecropin B (CB) has the most potent antibacterial properties and to examine the effects on cells and synthetic liposomes, CB derivatives cecropin B1 (CB1) and cecropin B3 (CB3) were developed. CB1 was made by replacing the C-terminal segment with CB's *N*-terminal sequence, while CB3 was made by replacing the *N*-terminal segment with CB's C-terminal sequence. CB and its equivalents have been shown in previous research to break membranes, and some of them can destroy cancer cells.

Preliminary SAR interpreting has been conducted using various methods such as molecular docking modelling and docking studies, where the interpretability is considerably better explicit. In this approach, pharmacophore techniques can provide a significant benefit and a clearer look at the structural elements that contribute to the structure activity relationship (SAR) [38]. In this study, pharmacophore generation was discovered using AutoDock vina 4 (The Scripps Research institute) [39] and discovery studio.

The scoring of ligands (binding affinity, ligand internal energy, and distance) is described in the result and discussion section. Using the default settings, the “protein-ligand interaction profiler” [40] calculated the binding affinity of ligands in stable ligand-protein complexes required for ligand binding with the receptor.

The molecular docking study shows that the value of dielectric constant is −**0.1465** and the binding spacing is **0.375.** The default setting of exhaustiveness is **8,** and the RMSD values are calculated relative to the best mode and use only movable heavy atoms. Two variants of RMSD metrics are provided, rmsd/lb (RMSD lower bound) and rmsd/ub (RMSD upper bound). In addition to this, the energy of binding modes in the output is 4. The “protein-ligand interaction profiler” (PLIP) was used to calculate the interaction of protein and ligands in the stable ligand-protein complex required for ligand binding to the receptor.

Based on molecular docking study of four compounds having thiopyrano[2,3-*b*]quinoline core **1**–**4** against **CB1a** (PDB ID: 2IGR). The docking result is given in Table 1.

In general, the IC_50_ of CB1a, CB, melittin, magainin II, as well as the cancer chemotherapeutic agent were determined using cytotoxicity assays on cancer and noncancer cells. CB1a has a stronger cytotoxic activity (lower IC_50_ values) against leukaemia cells and stomach cancer than CB and magainin II. CB1a and CB are highly effective towards cancerous cells but not normal human cells. CB1a has an 8-fold and 2-3-fold higher cytotoxic activity than CB against AGS and leukaemia cell lines, respectively. As a result, molecular docking study of the synthesized compounds was performed in the current study to explore their binding pattern with a unique anticancer peptide derived from the natural antimicrobial peptide cecropin B (CB1).

Because of its excellent selectivity, CB1a can be developed as a powerful anticancer drug. CB1a > CB1 > CB > CB3 is the order of anticancer activity of CB analogues against tumor cell lines [41]. Therefore, for this study, CB1a, a novel anticancer peptide derived from natural antimicrobial peptide cecropin B, was used. The synthetic compounds **(1**–**4)** were found to have binding affinity from −**5.3 to** −**6.1 kcal/mol** (Table 1) with the best result achieved using compound **4** (−**6.1 kcal/mol**). The hydrogen bond, residual interaction, and pi-pi interaction of the four compounds were summarized in (Table 1).

**Figure 3:**
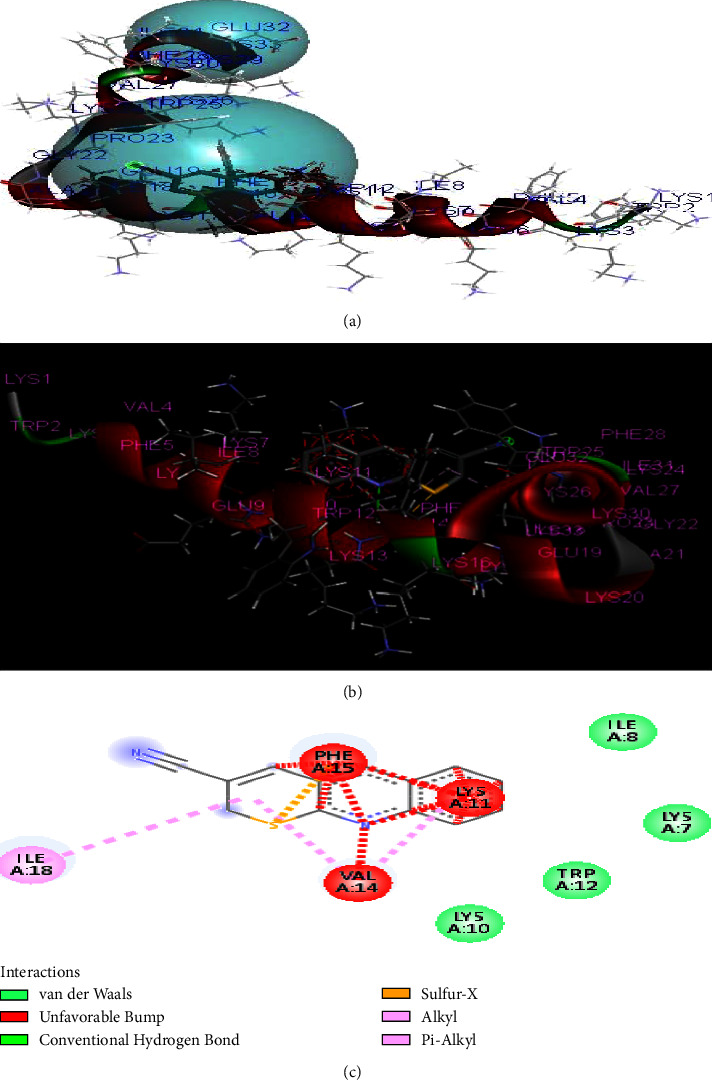
The binding interaction of thiopyrano[2,3-*b*]quinoline (**1**) with CB1a (PDB ID: 2IGR) (a) receptor cavities present during interaction, (b) interaction of protein and ligand, and (c) 2D-structure.

The compounds **(1)** show similar residual interactions with amino acid residues PHE-15, ILE A-8, ILE A-18, LYS A-7, LYS A-10, LYS A-11, VAL A-14, and TRP A-12 as shown in Figure 3. The *in-silico* interaction results match with the *in vitro* analysis of the synthesized compounds against the structure of CB1a, among which compound **1** shows low binding affinity as comparison to other derivatives with the value of −**5.3 kcal/mol**.

**Figure 4:**
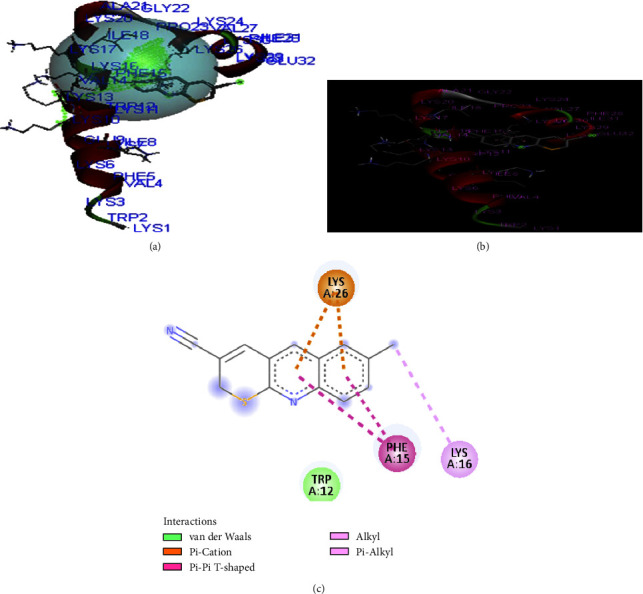
The binding interaction of 6-methyl-thiopyrano[2,3-*b*]quinoline (**2**) against CB1a (**PDB ID: 2IGR**) (a) receptor cavities present during interaction, (b) interaction of protein and ligand, and (c) 2D-structure.

The compounds **(2)** show similar residual interactions with amino acid residues PHE A-15, TRP A-12, LYS A-16, and LYS A-26 as shown in Figure 4. The data also show that there is additional hydrogen bonding interaction with amino acid residue GLU-32. The *in-silico* interaction results match the *in vitro* analysis of the synthesized compounds against the structure of CB1a. The binding affinity of compound **2** is found as −**5.5 kcal/mol**.

**Figure 5:**
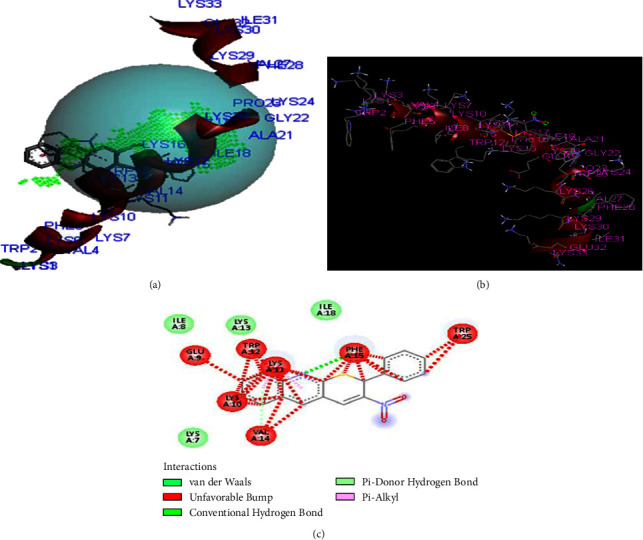
The binding interaction of 3-nitro-2-phenyl-2*H*-thiopyrano[2,3-*b*]quinoline (**3**) against CB1a (**PDB ID: 2IGR**) (a) receptor cavities present during interaction, (b) interaction of protein and ligand, and (c) 2D-structure.

The compounds **(3)** also show similar residual interactions with amino acid residues PHE A-15, ILE A-8, ILE A-18, LYS A-7, LYS A-10, LYS A-11, LYS A-13, VAL A-14, TRP A-12, TRP A-25, and GLU A-9 as shown in Figure 5. It shows additional hydrogen bonding with amino acid residue. The *in-silico* interaction results match the *in vitro* analysis of the synthesized compounds against the structure of CB1a, a novel anticancer peptide derived from natural antimicrobial peptide cecropin B among which compound **3** shows low binding affinity as comparison to other derivative and the value is −**5.9 kcal/mol**.

**Figure 6:**
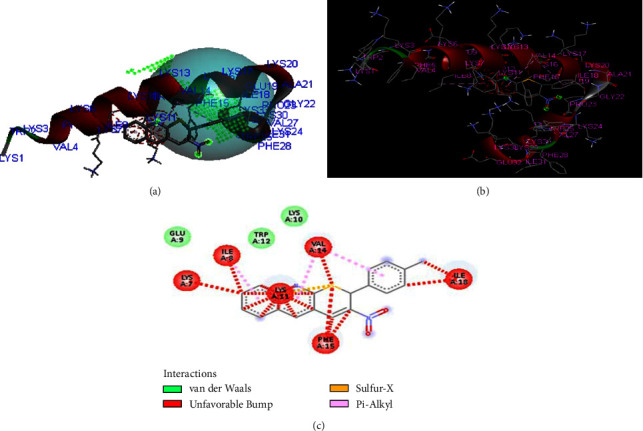
The binding interaction of 3-nitro-2-(4-chloro)phenyl-2*H*-thiopyrano[2,3-*b*]quinoline (**4**) against CB1a (**PDB ID: 2IGR**) (a) receptor cavities present during interaction, (b) interaction of protein and ligand, and (c) 2D-structure.

The compounds **(4)** show similar residual interactions with amino acid residues PHE A-15, ILE A-8, ILE A-18, LYS A-7, LYS A-11, VAL A-14, LYS A-16, LYS A-26, LYS A-10, and GLU A-9 as shown in Figure 6. The *in-silico* interaction of compound **4** results matches the *in vitro* analysis of the synthesized compounds against the structure of CB1a, which shows high binding affinity and good activity as compared to other derivatives since the value is −**6.1 kcal/mol**.

## 4. Conclusion

The current work demonstrates a useful study to find the possible effective antitumor agents having thiopyrano[2,3-*b*]quinoline derivatives, which could produce potent anticancer medicines in near future. To study the interaction of ligand and protein, several software packages were used in this work such as AutoDock vina 4, discovery studio, and protein-ligand interaction profiler (PLIP). The molecular docking of compounds having thiopyrano[2,3-*b*]quinoline core and its derivatives against CB1a (**PDB ID: 2IGR**) were studied in this article. The *in silico* molecular docking study of these compounds reveals that these frameworks have potential bioactivity having high binding affinity, adequate residual interaction, and hydrogen bonding interaction against the protein CB1a. The binding affinity value for thiopyrano[2,3-*b*]quinoline and its derivatives **1**–**4** is −**5.3 to** −**6.1 Kcal/mol**. The study also revealed that several amino acids show interaction with thiopyrano[2,3-*b*]quinoline ligand and their analogues. Some of the identified amino acids are ILE-8, ILE-18, LYS-7, LYS-11, VAL-14, LYS-16, LYS-26, PHE-15, LYS-15, TRP-12, TRP-25, LYS-10, and GLU-9. In general, the reported core already exhibits antibacterial activity and can be further refined to serve as anticancer compounds in near future.

## Figures and Tables

**Table 1:** Value of molecular docking of the compounds against CB1a.

Compound	Binding affinity (kcal/mol)	RMSD l.b.	RMSD u.b.	Hydrogen bonding interaction of amino acid (distance)	Hydrophobic interaction of amino acid (distance)	Pi-stacking	Pi-cation
1	−5.3	3.453	5.011	TRP A:12 (3.83), VAL A:14 (2.94)	PHE A:15 (3.63), LYS A:11 (3.93), ILE A:8 (3.71)	—	LYS A:11 (5.94)
2	−5.5	5.588	7.333	TRP A:12	LYS A:16	PHE A:15 (4.89)	LYS A:26 (3.28)
3	−5.9	2.458	3.269	GLU A:9 (3.17)	PHE A:15 (3.61), LYS A:11 (3.49), TRP A:25 (3.72), TRP A:12 (3.82), LYS A:10, VAL A:14 (2.97)	—	—
4	−6.1	6.842	8.714	LYS A:7, GLU A:9, GLU A:32 (3.17)	PHE A:15 (3.64), ILE A:18 (2.54), ILE A:8 (3.39), LYS A:11 (1.02), VAL A:14 (2.99)	—	—

## Data Availability

The data are available at the Department of Applied Chemistry, Amity School of Applied Sciences, Amity University Madhya Pradesh, Gwalior, Madhya Pradesh-474 005, India.

## Abbreviations

WHO: World Health Organization
HIV: Human immunodeficiency virus
FDA: Food and Drug Administration
PDB: Protein data bank
PLIP: Protein-ligand interaction profiler
TEA: Triethylamine
DMF: Dimethyl formamide
SAR: Structure activity relationship
RMSD: Root mean square deviation.

## References

[1] Nepali K., Sharma S., Sharma M., Bedi P. M. S., Dhar K. L. (2014). Rational approaches, design strategies, structure activity relationship and mechanistic insights for anticancer hybrids. *European Journal of Medicinal Chemistry*.

[2] Ćaleta I., Kralj M., Marjanović M. (2009). Novel Cyano- and amidinobenzothiazole derivatives: synthesis, antitumor evaluation, and X-ray and quantitative structure-activity relationship (QSAR) analysis. *Journal of Medicinal Chemistry*.

[3] Anttila S., Boffetta P. (2014). Occupational cancers. *Occupational Cancers*.

[4] Arbyn M., Weiderpass E., Bruni L. (2020). Estimates of incidence and mortality of cervical cancer in 2018: a worldwide analysis. *Lancet Global Health*.

[5] Ordikhani F., Erdem Arslan M., Marcelo R. (2016). Drug delivery approaches for the treatment of cervical cancer. *Pharmaceutics*.

[6] Chrysostomou A. C., Stylianou D. C., Constantinidou A., Kostrikis L. G. (2018). Cervical cancer screening programs in Europe: the transition towards HPV vaccination and population-based HPV testing. *Viruses*.

[7] Gatumo M., Gacheri S., Sayed A. R., Scheibe A. (2018). Women’s knowledge and attitudes related to cervical cancer and cervical cancer screening in Isiolo and Tharaka Nithi counties, Kenya: a cross-sectional study. *BMC Cancer*.

[8] DeBerardinis R. J., Lum J. J., Hatzivassiliou G., Thompson C. B. (2008). The biology of cancer: metabolic reprogramming fuels cell growth and proliferation. *Cell Metabolism*.

[9] Mandewale M. C., Patil U. C., Shedge S. V., Dappadwad U. R., Yamgar R. S. (2017). A review on quinoline hydrazone derivatives as a new class of potent antitubercular and anticancer agents. *Beni-Suef University Journal of Basic and Applied Sciences*.

[10] Baikar S., Malpathak N. (2010). Secondary metabolites as DNA topoisomerase inhibitors: a new era towards designing of anticancer drugs. *Pharmacognosy Reviews*.

[11] Canel C., Moraes R. M., Dayan F. E., Ferreira D. (2000). Phytochemistry. *Phytochemistry*.

[12] Pang X. C., Zhang L., Du G. H. (2018). *Natural Small Molecule Drugs from Plants*.

[13] Xu M., Wagerle T., Long J. K., Lahm G. P., Barry J. D., Smith R. M. (2014). Insecticidal quinoline and isoquinoline isoxazolines. *Bioorganic & Medicinal Chemistry Letters*.

[14] Eswaran S., Adhikari A. V., Shetty N. S. (2009). Synthesis and antimicrobial activities of novel quinoline derivatives carrying 1, 2, 4-triazole moiety. *European Journal of Medicinal Chemistry*.

[15] Foley M., Tilley L. (1998). Quinoline antimalarials: mechanisms of action and resistance and prospects for new agents. *Pharmacology & Therapeutics*.

[16] Hayat F., Salahuddin A., Umar S., Azam A. (2010). Synthesis, characterization, antiamoebic activity and cytotoxicity of novel series of pyrazoline derivatives bearing quinoline tail. *European Journal of Medicinal Chemistry*.

[17] Kumar Gupta S., Mishra A. (2016). Synthesis, characterization & screening for anti-inflammatory & analgesic activity of quinoline derivatives bearing azetidinones scaffolds. *Anti-Inflammatory & Anti-Allergy Agents in Medicinal Chemistry*.

[18] Ferlin M. G., Chiarelotto G., Antonucci F., Caparrotta L., Froldi G. (2002). Mannich bases of 3H-pyrrolo[3, 2-*f*]quinoline having vasorelaxing activity. *European Journal of Medicinal Chemistry*.

[19] Nikookar H., Mohammadi-Khanaposhtani M., Imanparast S. (2018). Design, synthesis and in vitro *α*-glucosidase inhibition of novel dihydropyrano[3, 2-*c*]quinoline derivatives as potential anti-diabetic agents. *Bioorganic Chemistry*.

[20] Singh S., Kaur G., Mangla V., Gupta M. K. (2015). Quinoline and quinolones: promising scaffolds for future antimycobacterial agents. *Journal of Enzyme Inhibition and Medicinal Chemistry*.

[21] Afzal O., Kumar S., Haider M. R. (2015). A review on anticancer potential of bioactive heterocycle quinoline. *European Journal of Medicinal Chemistry*.

[22] Tseng C. H., Tung C. W., Wu C. H. (2017). Discovery of indeno[1, 2-*c*]quinoline derivatives as potent dual antituberculosis and anti-inflammatory agents. *Molecules*.

[23] Muruganantham N., Sivakumar R., Anbalagan N., Gunasekaran V., Leonard J. T. (2004). Synthesis, anticonvulsant and antihypertensive activities of 8-substituted quinoline derivatives. *Biological and Pharmaceutical Bulletin*.

[24] Sashidhara K. V., Avula S. R., Mishra V. (2015). Identification of quinoline-chalcone hybrids as potential antiulcer agents. *European Journal of Medicinal Chemistry*.

[25] Ahmed N., Brahmbhatt K. G., Sabde S., Mitra D., Singh I. P., Bhutani K. K. (2010). Synthesis and anti-HIV activity of alkylated quinoline 2, 4-diols. *Bioorganic & Medicinal Chemistry*.

[26] Khan S. A., Asiri A. M., Basisi H. M. (2019). Synthesis and evaluation of Quinoline-3-carbonitrile derivatives as potential antibacterial agents. *Bioorganic Chemistry*.

[27] Khan S. A., Razvi M. A. N., Bakry A. H., Afzal S. M., Asiri A. M., El-Daly S. A. (2015). Microwave assisted synthesis, spectroscopic studies and non linear optical properties of bis-chromophores. *Spectrochimica Acta Part A: Molecular and Biomolecular Spectroscopy*.

[28] Khan S. A. (2017). Green synthesis, spectrofluorometric characterization and antibacterial activity of heterocyclic compound from chalcone on the basis of in vitro and quantum chemistry calculation. *Journal of Fluorescence*.

[29] Khan S. A., Ullah Q., Syed S. (2021). Microwave assisted one-pot synthesis, photophysical and physicochemical studies of novel biologically active heterocyclic Donor (D)-*π*-Acceptor (A) chromophore. *Bioorganic Chemistry*.

[30] Pommier Y. (2006). Topoisomerase I inhibitors: camptothecins and beyond. *Nature Reviews Cancer*.

[31] Alqasoumi S. I., Al-Taweel A. M., Alafeefy A. M., Hamed M. M., Noaman E., Ghorab M. M. (2009). Synthesis and biological evaluation of 2-amino-7, 7-dimethyl 4-substituted-5-oxo-1-(3, 4, 5-trimethoxy)-1, 4, 5, 6, 7, 8-hexahydro-quinoline-3-carbonitrile derivatives as potential cytotoxic agents. *Bioorganic & Medicinal Chemistry Letters*.

[32] Ghorab M. M., Ragab F. A., Heiba H. I., Ghorab W. M. (2011). Design and synthesis of some novel quinoline derivatives as anticancer and radiosensitizing agents targeting VEGFR tyrosine kinase. *Journal of Heterocyclic Chemistry*.

[33] Katariya K. D., Shah S. R., Reddy D. (2020). Anticancer, antimicrobial activities of quinoline based hydrazone analogues: synthesis, characterization and molecular docking. *Bioorganic Chemistry*.

[34] Meth-Cohn O., Narine B., Tarnowski B. (1981). A versatile new synthesis of quinolines and related fused pyridines, Part 5. The synthesis of 2-chloroquinoline-3-carbaldehydes. *Journal of the Chemical Society, Perkin Transactions 1*.

[35] Srivastava A., Singh R. M. (2006). Vilsmeier-Haack reagent: a facile synthesis of 2-chloro-3-formylquinolines from N-arylacetamides and transformation into different functionalities. *ChemInform*.

[36] Singh B., Chandra A., Asthana M., Singh R. M. (2012). Rapid, clean and efficient one-pot synthesis of thiopyrano[2, 3-*b*]quinolines via domino Michael addition/cyclization reactions. *Tetrahedron Letters*.

[37] Kumar S. V., Muthusubramanian S., Perumal S. (2015). Facile “on water” domino reactions for the expedient synthesis of 2*H*-thiopyrano[2, 3-*b*]quinolines. *RSC Advances*.

[38] Fayed E. A., Eissa S. I., Bayoumi A. H., Gohar N. A., Mehany A. B. M., Ammar Y. A. (2019). Design, synthesis, cytotoxicity and molecular modeling studies of some novel fluorinated pyrazole-based heterocycles as anticancer and apoptosis-inducing agents. *Molecular Diversity*.

[39] Trott O., Olson A. J. (2010). AutoDock Vina: improving the speed and accuracy of docking with a new scoring function, efficient optimization and multithreading. *Journal of Computational Chemistry*.

[40] Salentin S., Schreiber S., Haupt V. J., Adasme M. F., Schroeder M. (2015). PLIP: fully automated protein–ligand interaction profiler. *Nucleic Acids Research*.

[41] Wu J. M., Jan P. S., Yu H. C. (2009). Structure and function of a custom anticancer peptide, CB1a. *Peptides*.

