# Supplementary material for: Molecular Docking Study for Binding Affinity of 2H-thiopyrano[2,3-b]quinoline Derivatives against CB1a
Source: Interdiscip Perspect Infect Dis. 2023 Jan 9;2023:1618082. doi: 10.1155/2023/1618082 (PMC9842416; doi:10.1155/2023/1618082)
Supplement: Supplementary Materials — All the interaction details and necessary data are available at the supplementary data. [file 1618082.f1.docx]

**Molecular Docking Analysis and Anticancer Activity of 2*H*-thiopyrano[2,3-*b*]quinoline derivatives against CB1a**

Shivangi Sharma and Shivendra Singh*

[shivangiksharma1997@gmail.com](mailto:shivangiksharma1997@gmail.com) and [ssingh4@gwa.amity.edu](mailto:ssingh4@gwa.amity.edu)

Department of Applied Chemistry, Amity School of Applied Sciences, Amity University Madhya Pradesh, Gwalior, Madhya Pradesh-474 005, India

| **Compound** | **Binding affinity (kcal/mol)** | **RMSD l.b.** | **RMSD u.b.** | **Hydrogen bonding interaction of Amino acid (distance)** | **Hydrophobic interaction of Amino acid (distance)** | **Pi-stacking** | **Pi-cation** |  |
| --- | --- | --- | --- | --- | --- | --- | --- | --- |
|  |  |  |  |  |  |  |  |  |
| **1** | -5.3 | 3.453 | 5.011 | TRP A:12(3.83), VAL A:14(2.94), | PHE A:15(3.63), LYS A:11(3.93), ILE A:8(3.71) | **-** | LYS A:11 (5.94) |  |
| **2** | -5.5 | 5.588 | 7.333 | TRP A:12, | LYS A:16 | PHE A:15(4.89) | LYS A:26 (3.28) |  |
| **3** | -5.9 | 2.458 | 3.269 | GLU A:9(3.17) | PHE A:15(3.61), LYS A:11(3.49), TRP A:25(3.72), TRP A:12(3.82), LYS A:10, VAL A:14(2.97) | **-** | **-** |  |
| **4** | -6.1 | 6.842 | 8.714 | LYS A:7, GLU A:9, GLU A:32 (3.17) | PHE A:15(3.64), ILE A:18(2.54), ILE A: 8(3.39), LYS A:11(1.02), VAL A:14(2.99) | **-** | **-** |  |
|  |  |  |  |  |  |  |  |  |

**1.     The binding interaction of thiopyrano[2,3-b]quinoline (1) with CB1a (PDB ID: 2IGR).**

**For hydrogen bonding-**

| **RESNR** | **RESTYPE** | **RESCHAIN** | **RESNR_LIG** | **RESTYPE_LIG** | **RESCHAIN_LIG** | **SIDECHAIN** | **DIST_H-A** | **DIST_D-A** | **DON_ANGLE** | **PROTISDON** | **DONORIDX** | **DONORTYPE** | **ACCEPTORIDX** | **ACCEPTORTYPE** | **LIGCOO** | **PROTCOO** |
| --- | --- | --- | --- | --- | --- | --- | --- | --- | --- | --- | --- | --- | --- | --- | --- | --- |
| **12** | TRP | A | 1 | LIG | Z | True | 3.13 | 3.83 | 129.44 | True | 162 | Nar | 1 | N1 | -2.505, -1.514, 7.707 | -5.750, 0.417, 7.089 |
| **26** | LYS | A | 1 | LIG | Z | True | 2.62 | 3.39 | 130.96 | True | 314 | N3+ | 1 | N1 | -2.505, -1.514, 7.707 | -1.172, -0.898, 10.766 |

**For hydrophobic interaction-**

| **RESNR** | **RESTYPE** | **RESCHAIN** | **RESNR_LIG** | **RESTYPE_LIG** | **RESCHAIN_LIG** | **DIST** | **LIGCARBONIDX** | **PROTCARBONIDX** | **LIGCOO** | **PROTCOO** |
| --- | --- | --- | --- | --- | --- | --- | --- | --- | --- | --- |
| **8** | ILE | A | 1 | LIG | Z | 3.71 | 7 | 113 | -1.992, 5.206, 3.075 | -4.882, 7.427, 2.410 |
| **8** | ILE | A | 1 | LIG | Z | 3.57 | 8 | 112 | -1.362, 6.336, 2.558 | -3.412, 8.700, 0.840 |
| **11** | LYS | A | 1 | LIG | Z | 3.54 | 7 | 144 | -1.992, 5.206, 3.075 | -1.091, 2.860, 0.579 |
| **11** | LYS | A | 1 | LIG | Z | 3.93 | 8 | 145 | -1.362, 6.336, 2.558 | -0.040, 3.750, -0.088 |
| **15** | PHE | A | 1 | LIG | Z | 3.63 | 13 | 196 | -1.307, 0.497, 6.916 | 0.345, -1.273, 4.215 |

**For Pi-cation-**

| **RESNR** | **RESTYPE** | **RESCHAIN** | **PROT_IDX_LIST** | **RESNR_LIG** | **RESTYPE_LIG** | **RESCHAIN_LIG** | **DIST** | **OFFSET** | **PROTCHARGED** | **LIG_GROUP** | **LIG_IDX_LIST** | **LIGCOO** | **PROTCOO** |
| --- | --- | --- | --- | --- | --- | --- | --- | --- | --- | --- | --- | --- | --- |
| **11** | LYS | A | 147 | 1 | LIG | Z | 5.94 | 1.66 | True | Aromatic | 3,4,5,6,7,8 | -0.665, 5.463, 3.409 | 1.806, 3.775, -1.723 |

**2.     The binding interaction of 6-methyl-thiopyrano[2,3-b]quinoline (2) against CB1a (PDB ID: 2IGR).**

**For hydrogen bonding-**

| **RESNR** | **RESTYPE** | **RESCHAIN** | **RESNR_LIG** | **RESTYPE_LIG** | **RESCHAIN_LIG** | **SIDECHAIN** | **DIST_H-A** | **DIST_D-A** | **DON_ANGLE** | **PROTISDON** | **DONORIDX** | **DONORTYPE** | **ACCEPTORIDX** | **ACCEPTORTYPE** | **LIGCOO** | **PROTCOO** |
| --- | --- | --- | --- | --- | --- | --- | --- | --- | --- | --- | --- | --- | --- | --- | --- | --- |
| **26** | LYS | A | 1 | LIG | Z | TRUE | 3.35 | 3.87 | 112.08 | True | 298 | N3+ | 385 | N3 | -0.178, 1.085, 7.597 | -1.172, -0.898, 10.766 |
| **32** | GLU | A | 34 | NH2 | A | False | 2.52 | 3.17 | 124.09 | False | 382 | N3 | 361 | O2 | 3.155, -3.972, 25.011 | 3.845, -1.300, 23.442 |

**For hydrophobic interaction**

| **RESNR** | **RESTYPE** | **RESCHAIN** | **RESNR_LIG** | **RESTYPE_LIG** | **RESCHAIN_LIG** | **DIST** | **LIGCARBONIDX** | **PROTCARBONIDX** | **LIGCOO** | **PROTCOO** |
| --- | --- | --- | --- | --- | --- | --- | --- | --- | --- | --- |
| **25** | TRP | A | 1 | LIG | Z | 3.3 | 393 | 278 | 1.456, -1.114, 8.117 | 3.671, -3.492, 7.565 |

**For Pi-stacking**

| **RESNR** | **RESTYPE** | **RESCHAIN** | **PROT_IDX_LIST** | **RESNR_LIG** | **RESTYPE_LIG** | **RESCHAIN_LIG** | **DIST** | **OFFSET** | **PROTCHARGED** | **LIG_GROUP** | **LIG_IDX_LIST** | **LIGCOO** | **PROTCOO** |
| --- | --- | --- | --- | --- | --- | --- | --- | --- | --- | --- | --- | --- | --- |
| **15** | PHE | A | 1 | LIG | Z | 178, 179, 180, 181, 182, 183 | 4.89 | 79.5 | 0.94 | T | 386, 387, 388, 389, 390, 391 | 0.120, -0.721, 7.757 | 0.764, -0.969, 2.915 |

**For Pi-cation**

| **RESNR** | **RESTYPE** | **RESCHAIN** | **PROT_IDX_LIST** | **RESNR_LIG** | **RESTYPE_LIG** | **RESCHAIN_LIG** | **DIST** | **OFFSET** | **PROTCHARGED** | **LIG_GROUP** | **LIG_IDX_LIST** | **LIGCOO** | **PROTCOO** |
| --- | --- | --- | --- | --- | --- | --- | --- | --- | --- | --- | --- | --- | --- |
| **26** | LYS | A | 298 | 1 | LIG | Z | 3.28 | 0.33 | True | Aromatic | 386, 387, 388, 389, 390, 391 | 0.120, -0.721, 7.757 | -1.172, -0.898, 10.766 |

**3.     The binding interaction of 3-nitro-2-phenyl-2H-thiopyrano[2,3-b]quinoline (3) against CB1a (PDB ID: 2IGR).**

**For hydrogen bonding-**

| **RESNR** | **RESTYPE** | **RESCHAIN** | **RESNR_LIG** | **RESTYPE_LIG** | **RESCHAIN_LIG** | **SIDECHAIN** | **DIST_H-A** | **DIST_D-A** | **DON_ANGLE** | **PROTISDON** | **DONORIDX** | **DONORTYPE** | **ACCEPTORIDX** | **ACCEPTORTYPE** | **LIGCOO** | **PROTCOO** |
| --- | --- | --- | --- | --- | --- | --- | --- | --- | --- | --- | --- | --- | --- | --- | --- | --- |
| **32** | GLU | A | 34 | NH2 | A | False | 2.52 | 3.17 | 382 | False | 298 | N3 | 361 | O2 | 3.155, -3.972, 25.011 | 3.845, -1.300, 23.442 |

**For hydrophobic interaction**

| **RESNR** | **RESTYPE** | **RESCHAIN** | **RESNR_LIG** | **RESTYPE_LIG** | **RESCHAIN_LIG** | **DIST** | **LIGCARBONIDX** | **PROTCARBONIDX** | **LIGCOO** | **PROTCOO** |
| --- | --- | --- | --- | --- | --- | --- | --- | --- | --- | --- |
| **11** | LYS | A | 1 | LIG | Z | 3.49 | 403 | 128 | 0.728, 2.682, 3.548 | -1.091, 2.860, 0.579 |
| **12** | TRP | A | 1 | LIG | Z | 3.82 | 388 | 145 | -9.147, 5.309, 6.233 | -8.807, 1.537, 5.704 |
| **12** | TRP | A | 1 | LIG | Z | 3.72 | 390 | 141 | -6.771, 4.886, 5.914 | -6.259, 1.289, 5.106 |
| **15** | PHE | A | 1 | LIG | Z | 3.61 | 403 | 183 | 0.728, 2.682, 3.548 | 2.081, -0.552, 2.684 |

**4.     The binding interaction of 3-chloro-3-nitro-2-phenyl-2H-thiopyrano[2,3-b]quinoline (4) against CB1a (PDB ID: 2IGR).**

**For hydrogen bonding-**

| **RESNR** | **RESTYPE** | **RESCHAIN** | **RESNR_LIG** | **RESTYPE_LIG** | **RESCHAIN_LIG** | **SIDECHAIN** | **DIST_H-A** | **DIST_D-A** | **DON_ANGLE** | **PROTISDON** | **DONORIDX** | **DONORTYPE** | **ACCEPTORIDX** | **ACCEPTORTYPE** | **LIGCOO** | **PROTCOO** |
| --- | --- | --- | --- | --- | --- | --- | --- | --- | --- | --- | --- | --- | --- | --- | --- | --- |
| **32** | GLU | A | 34 | NH2 | A | False | 2.52 | 3.17 | 124.09 | False | 647 | N3 | 612 | O2 | 3.155, -3.972, 25.011 | 3.845, -1.300, 23.442 |

**For hydrophobic interaction**

| **RESNR** | **RESTYPE** | **RESCHAIN** | **RESNR_LIG** | **RESTYPE_LIG** | **RESCHAIN_LIG** | **DIST** | **LIGCARBONIDX** | **PROTCARBONIDX** | **LIGCOO** | **PROTCOO** |
| --- | --- | --- | --- | --- | --- | --- | --- | --- | --- | --- |
| **8** | ILE | A | 1 | LIG | Z | 3.39 | 657 | 156 | -2.074, 5.670, 0.117 | -3.412, 8.700, 0.840 |
| **11** | LYS | A | 1 | LIG | Z | 0.57 | 654 | 211 | -2.099, 2.850, 0.117 | -2.485, 3.272, 0.105 |
| **11** | LYS | A | 1 | LIG | Z | 1.02 | 658 | 213 | 0.310, 2.814, 0.116 | -0.040, 3.750, -0.088 |
| **14** | VAL | A | 1 | LIG | Z | 2.99 | 660 | 280 | -0.991, 0.813, 0.116 | -2.933, -1.163, -1.020 |
| **18** | ILE | A | 1 | LIG | Z | 2.36 | 667 | 361 | 1.074, -4.618, 1.318 | 0.155, -6.044, 2.956 |
| **18** | ILE | A | 1 | LIG | Z | 2.54 | 666 | 359 | 1.125, -5.568, 0.298 | 0.331, -7.247, 2.027 |

**Thank You**

**Dr. Shivendra Singh**

**Assistant Professor-II**
